# Cognitive impairment assessment through handwriting (COGITAT) score: a novel tool that predicts cognitive state from handwriting for forensic and clinical applications

**DOI:** 10.3389/fpsyg.2024.1275315

**Published:** 2024-03-28

**Authors:** Maurizio Balestrino, Andrea Brugnolo, Nicola Girtler, Matteo Pardini, Cristiano Rizzetto, Paolo Alessandro Alì, Leonardo Cocito, Irene Schiavetti

**Affiliations:** ^1^Department of Neuroscience, Rehabilitation, Ophthalmology, Genetics and Maternal and Child Sciences (DINOGMI), University of Genoa, Genoa, Italy; ^2^IRCCS Ospedale Policlinico San Martino, Genoa, Italy; ^3^Department of Health Sciences (DISSAL), University of Genoa, Genoa, Italy

**Keywords:** forensic science, will challenge, handwriting analysis, cognitive impairment, dementia, posthumous

## Abstract

**Introduction:**

Handwriting deteriorates proportionally to the writer’s cognitive state. Such knowledge is of special importance in the case of a contested will, where dementia of the testator is claimed, but medical records are often insufficient to decide what the testator’s cognitive state really was. By contrast, if the will is handwritten, handwriting analysis allows us to gauge the testator’s cognitive state at the precise moment when he/she was writing the will. However, quantitative methods are needed to precisely evaluate whether the writer’s cognitive state was normal or not. We aim to provide a test that quantifies handwriting deterioration to gauge a writer’s cognitive state.

**Methods:**

We consecutively enrolled patients who came for the evaluation of cognitive impairment at the Outpatient Clinic for Cognitive Impairment of the Department of Neuroscience, Rehabilitation, Ophthalmology, Genetics and Maternal and Child Sciences (DINOGMI) of the University of Genoa, Italy. Additionally, we enrolled their caregivers. We asked them to write a short text by hand, and we administered the Mini Mental State Examination (MMSE). Then, we investigated which handwriting parameters correlated with cognitive state as gauged by the MMSE.

**Results:**

Our study found that a single score, which we called the **COG**nitive **I**mpairment **T**hrough h**A**ndwri**T**ing (**COGITAT**) score, reliably allows us to predict the writer’s cognitive state.

**Conclusion:**

The COGITAT score may be a valuable tool to gage the cognitive state of the author of a manuscript. This score may be especially useful in contested handwritten wills, where clinical examination of the writer is precluded.

## 1 Introduction

Available scientific data indicate that cognitive impairment significantly compromises handwriting. Already in 1911 Tamburini claimed, citing the works of his contemporaries Borri and Grilli, that in patients diagnosed with dementia, affected by what was then known as progressive general paralysis, “the handwriting had that special character which alone betrays the profound neuro-psychic alteration of the writer” (Tamburini, 1911). Coming to current times, healthy subjects make spelling mistakes in 2% of words versus 25% of patients with mild Alzheimer’s disease and 83% of patients with severe Alzheimer’s disease (Silveri et al., 2007). The handwriting of Alzheimer’s disease patients worsens as the disease progresses (Luzzatti et al., 2003) and indeed the simple inspection of a handwritten sentence gives some hints on the cognitive status of the person who wrote it (Shenkin et al., 2008). Alzheimer’s disease patients produce shorter and less informative writings, produce more paraphasias, and make more mistakes in letter formation (Forbes et al., 2004). There are strong and marked correlations between cognitive tests and parameters of handwriting such as the length of the text, the number of comprehensible words and the amount of errors (Renier et al., 2016). Lexical, semantic and syntactic parameters of the written text, as well as frequency of spelling errors, are not impaired by normal aging but they are by Alzheimer’s disease (Croisile, 2005).

Analysis of handwriting to ascertain a possible cognitive impairment is of special importance in the case of a contested will, where dementia of the testator is often claimed. In such trials, medical records are often insufficient, and witnesses often offer contradictory or unreliable reports. By contrast, analysis of handwriting offers the possibility of gauging the testator’s performance in the precise moment when he/she was making the will. A poor handwriting may then indicate a cognitive impairment.

To carry out such an analysis one needs a score that quantifies handwriting deterioration, and a cutoff for cognitive impairment. To this aim, we created and investigated the “writing score,” which quantifies how much handwriting is compromised (Fontana et al., 2008; Balestrino et al., 2012). The “writing score” evaluates, in a semi-quantitative manner, the legibility of the text as well as its spatial orientation. We demonstrated (Fontana et al., 2008; Balestrino et al., 2012) that the writing score correlates significantly with both the Mini Mental State Examination (Folstein et al., 1975) and the Milan Overall Dementia Assessment (Brazzelli et al., 1994). Its predictive value is rather reliable for scores at either end of its scale; very low scores predict cognitive impairment while very high scores predict cognitive normality. However, intermediate scores are not very specific, occurring both in cognitively compromised persons and in normal controls (Balestrino et al., 2017).

With the present research, we further investigate the relationship between handwriting and cognitive status, and we attempt to identify a cutoff score that may reliably identify subjects with cognitive impairment.

## 2 Methods

### 2.1 Patients’ enrollment

Patients were consecutively enrolled from those seeking clinical attention for cognitive impairment evaluation at the Outpatient Clinic for Cognitive Impairment of the Department of Neuroscience, Rehabilitation, Ophthalmology, Genetics and Maternal and Child Sciences (DINOGMI) of the University of Genoa, Italy. Caregivers accompanying them were also included in the investigation. To enhance sample representativeness for the general population, the only inclusion criteria were the absence of severe visual sensory deficits and Italian mother-tongue. Initially, all patients and caregivers underwent testing, but subsequently, all subjects (patients and caregivers) younger than 50-year-old were excluded. We selected this cutoff because caregivers were mostly in that age group, and 50 is the age when the earliest cases of cognitive impairment occur (Albert and Heaton, 1988). All subjects signed an institutional consent form before enrollment in the study. All participants provided written informed consent, agreeing to the use and processing of their data for scientific purposes. They received information about the study’s purpose, data usage, and their right to withdraw without affecting their clinical care. Ethical review and approval were not necessary for the study in compliance with national legislation and institutional requirements. The study was conducted in accordance with the national legislation and institutional requirements. The participants provided their written informed consent for research participation.

### 2.2 Neuropsychological and handwriting test

In addition to the usual assessment, which routinely includes an MMSE, all patients were asked to write a spontaneous text on blank paper. Caregivers were separately administered an MMSE and asked to write a spontaneous text as well. Both patients and caregivers were instructed as follows: “Write whatever you like on this paper, using no more than 6 or 7 lines. Do not worry about errors or corrections, this is not a school examination, and no one will give you a grade.”

**Figure 1:**
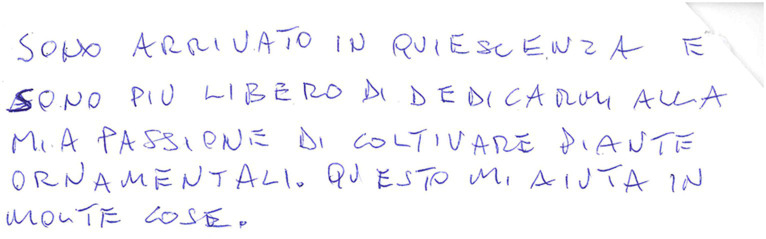
Example of a manuscript that was scored 5 (“normally oriented rows. In each row, beginning and end correspond to the page margins”) in the “Spatial orientation” item of the “Writing Score.”

**Figure 2:**
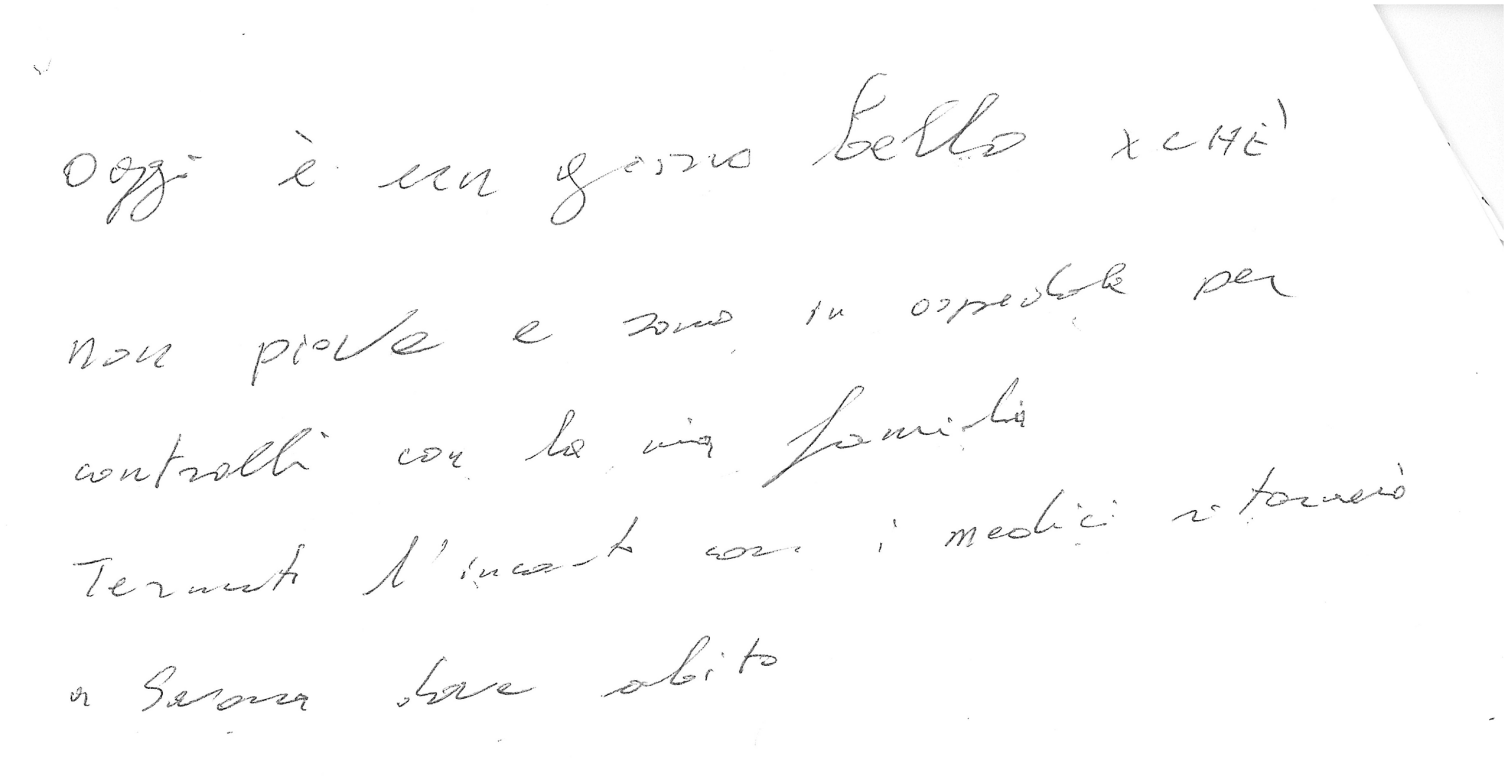
Example of a manuscript that was scored 4 (“rows slightly distorted or with beginning and end bearing little correspondence to the page margins”) in the “Spatial orientation” item of the “Writing Score.”

**Figure 3:**
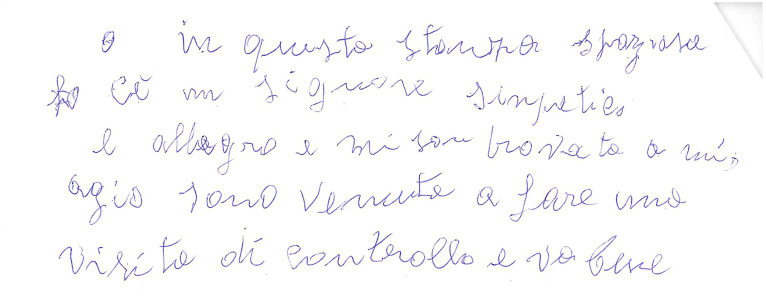
Example of a manuscript that was scored 4 (“rows slightly distorted or with beginning and end bearing little correspondence to the page margins”) in the “Spatial orientation” item of the “Writing Score.” Please note that in this case the score 4 was attributed because even if the rows are fairly horizontal the margins bear little correspondence to the page margins.

**Figure 4:**
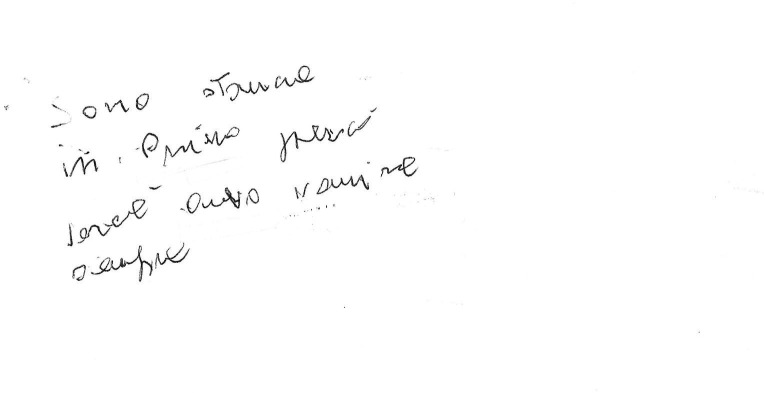
Example of a manuscript that was scored 3 (“rows clearly distorted or with beginning and end not corresponding to the page margins”) in the “Spatial orientation” item of the “Writing Score.” Please note that underwriting of some words was done by the examiners during handwriting analysis.

**Figure 5:**
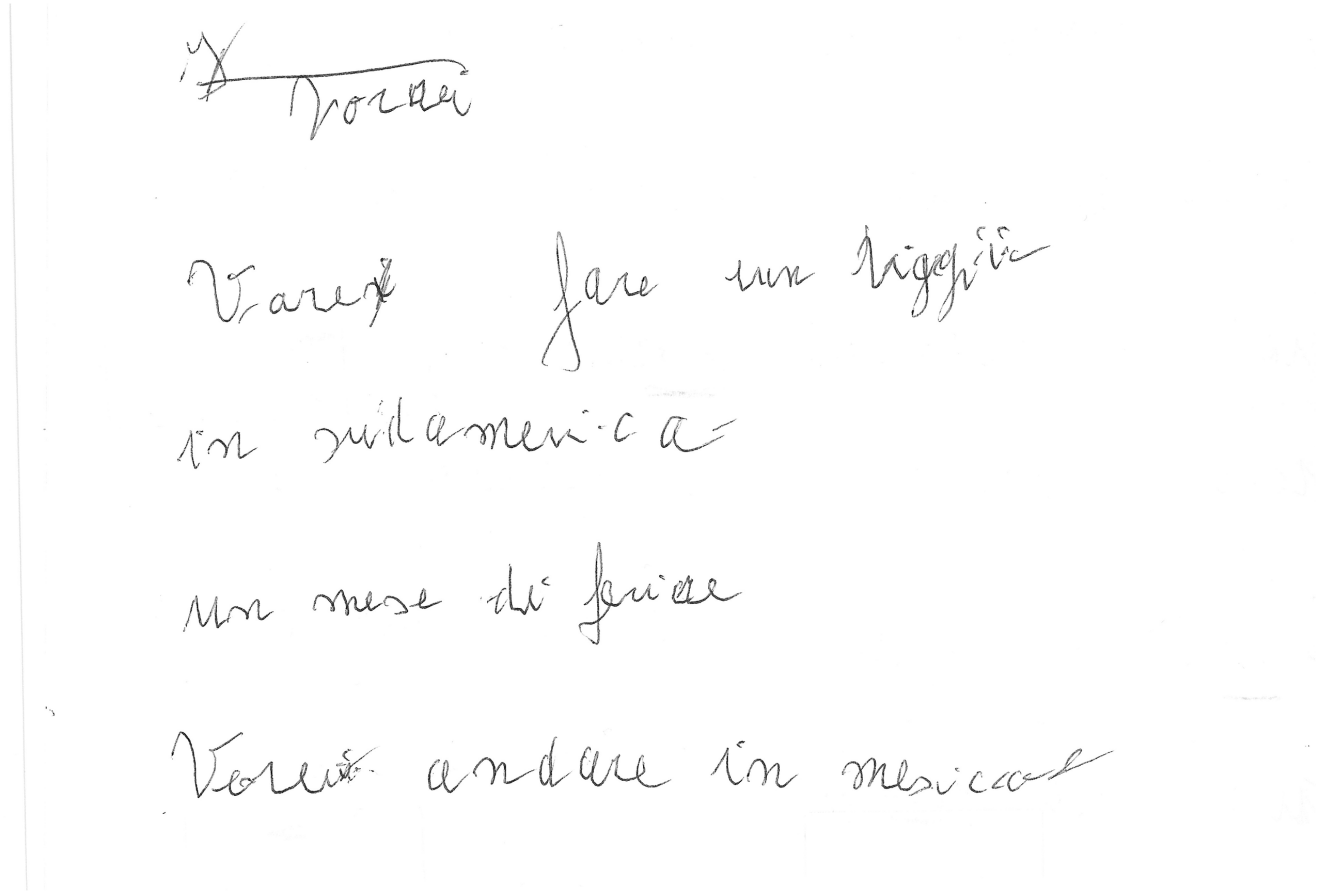
Example of a manuscript that was scored 3 (“rows clearly distorted or with beginning and end not corresponding to the page margins”) in the “Spatial orientation” item of the “Writing Score.” Please note that in this case the score 3 was attributed because even if the rows are fairly horizontal the margins are not corresponding to the page margins.

**Figure 6:**
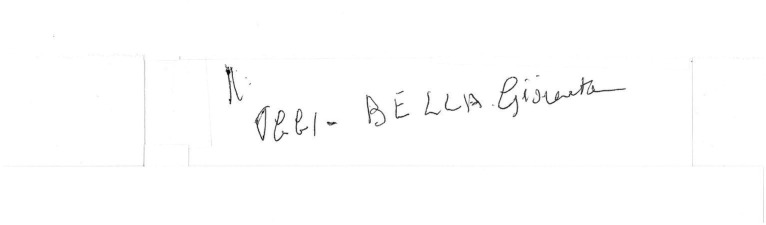
Example of a manuscript that was scored 2 (“Words or letters inserted where they do not belong in the text.”) in the “Spatial orientation” item of the “Writing Score.” Please note that in this example an indecipherable grapheme is placed out of context in the upper left corner.

**Figure 7:**
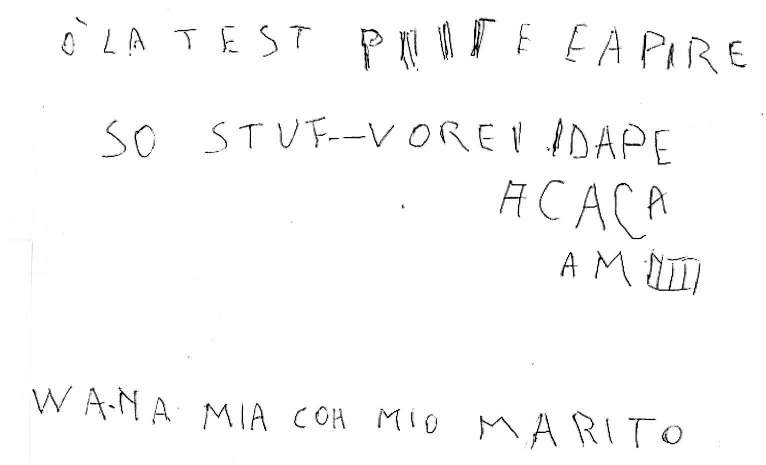
Example of a manuscript that was scored 1 (“chaotic orientation of the rows”) in the “Spatial orientation” item of the “Writing Score.”

For all handwriting samples, we assessed:

- The Writing Score (Fontana et al., 2008; Balestrino et al., 2012), a numerical measure of handwriting quality representing the sum of two scores. The first evaluates overall correctness and legibility from a verbal standpoint (the “Verbal and lexical skills” scale), while the second evaluates spatial orientation, specifically the horizontal alignment of lines and how closely margins correspond to those of the sheet (the “Spatial orientation” scale). Each scale ranges from 1 to 5, with higher score indicating better quality. Please refer to (Balestrino et al., 2012) for additional details and handwriting samples. In this manuscript, Figures 1–7 show handwriting samples illustrating various “Spatial orientation” scores. Moreover, Supplementary Table II illustrates the Writing Score, as originally published. Briefly, the “writing score” is a categorical, semiquantitative score, whose values are assigned based on the specific definitions that some of us ideated and published (Fontana et al., 2008; Balestrino et al., 2012). As such, it is not the result of measurements on the text, but it is the result of the evaluator’s judgment. We emphasize that the score obtained in this way correlates significantly with formal neuropsychological tests of cognitive state (*ibidem*), and that the test has a significant inter-observer agreement (Fontana et al., 2008). To the best of our knowledge, the writing score is the only quantitative method that allows evaluation of handwriting in a forensically relevant way.
- The percentage of spelling and grammar errors, defined as the percentage of words in the text containing such errors. We considered as “spelling and grammar errors” those resulting in a mistake in how the word is written, for example letters missing or replaced. We did not considered as errors letters traced in an incorrect way (e.g., a letter “t” missing the horizontal tract)
- The total number of words written
- The percentage of words written, even partially, in capital letters.

### 2.3 Statistical analysis

Based on their MMSE score, all subjects were categorized as normal (MMSE≥24) or with cognitive impairment (MMSE <24). This score was the cutoff that separated normal from demented people in a large validation study in Italy (Measso et al., 1993) and it is still largely used by Italian neurologists. This cutoff score is also one of the most widely used worldwide (Tsoi et al., 2015).

Baseline characteristics were reported as mean ± standard deviation (s.d.) or count with frequency, as appropriate.

A novel predictive score useful for identifying the probability of cognitive impairment was derived and validated through a univariate and subsequent multivariate stepwise logistic regression analyses.

The model considered demographic variables as well as all items collected for spontaneous text. We chose to use spontaneous testing in building the score because it is more easily recoverable, even in normal real-life conditions, compared to dictated text that would need to be requested specifically. Variables with value of *p* < 0.20 in the univariate model (age, years of education, writing score—verbal and lexical skills, writing score - spatial orientation, total number of words, and percent of error) were candidates for multivariate analysis, where a backward stepwise variable selection with a value of *p* < 0.10 for inclusion and exclusion was applied.

Coefficients (β) with their standard error (S.E.), together with Odds Ratio (OR) and 95% Confidence Interval (CI) were estimated for each of the significant variables. ROC curve was graphed for identifying the Area Under the Curve (AUC) and assessing the discrimination of the fitted logistic model.

The new score was validated with a split-sample internal validation method. The whole sample was randomly divided into two groups, a training cohort (70%) and an internal validation cohort (30%) based on random computer generation. Characteristics of patients in the two data sets were compared using the chi-square test or Fisher’s exact test for categorical variables and the Mann–Whitney U test for continuous variables.

The regression model applied to the whole group was firstly replicated on the training cohort to verify whether it produced the same subset of predictors. Coefficients (β) obtained from the regression analysis on the training cohort were used for deriving a score defined as the linear combination of the coefficients multiplied by the corresponding value of the n variables (score = β1 × var1 + β2 × var2 + … + βn × varn), where higher scores represented a greater risk for cognitive impairment.

The discriminating performance of the score was evaluated in two steps. Firstly, in the training dataset two optimal cut-off scores were identified by maximizing, respectively, their specificity and their sensitivity, so as to detect with approximately 95% probability those subjects that were or, respectively, were not cognitively impaired. Subsequently, the performance of the score was assessed in the validation sample by applying a univariable logistic regression model with the binary score and by deriving sensitivity, specificity and AUC with relating 95% CI.

The probability of showing cognitive impairment based on the estimated coefficients as follows:


$$\begin{array}{l} \mathrm{Probability}\;\mathrm{of}\;\mathrm{cognitive}\;\mathrm{impairment}\;\left(P\right)\\ \;=\frac{e^{\left(\beta 0+\beta 1\;\mathrm{var}1+\ldots +\beta n\;\mathrm{varn}\right)}}{1+e^{\left(\beta 0+\beta 1\;\mathrm{var}1+\ldots +\beta n\;\mathrm{varn}\right)}} \end{array}$$

The recommended sample-to-variable ratio suggests a minimum observation-to-variable ratio of 5:1, with preferred ratios of 15:1 or 20:1 (Hair et al., 2018). Consequently, for our internal validity study, which involves 11 independent variables in a logistic model, we have considered a minimum sample size of 165 patients (15:1), with a final enrollment of 167 patients.

Statistical analysis was performed using SPSS (RRID:SCR_002865) version 24.0.

## 3 Results

One hundred and sixty-seven adult individuals (patients and caregivers) aged 50 years old or older (range 50–93 years) were enrolled. Fifty-four of them (32.3%) had cognitive impairment (defined as MMSE<24), whereas the remaining 113 subjects (67.7%) reported a normal value of MMSE (≥ 24). Table 1 summarizes baseline data and identified scores and evaluations-related writing characteristics of the whole sample and for each MMSE group. The full database is included in the Supplementary material.

**Table 1:** Baseline characteristics of enrolled subjects (*N* = 167).

	Normal (MMSE ≥ 24)	Cognitive impairment (MMSE < 24)	Total
	*n* = 113	*n* = 54	*n* = 167
Sex, males—*n (%)*	41 (36.3)	17 (31.5)	58 (34.7)
Age (years)—*mean ± s.d.*	68.5 ± 11.62	79.2 ± 8.38	72.0 ± 11.78
Years of education—*mean ± s.d.*	12.1 ± 4.81	8.2 ± 3.86	10.8 ± 4.86
MMSE—*mean ± s.d.*	28.4 ± 1.89	17.1 ± 4.45	24.8 ± 6.07
Spontaneous Text			
Total writing score—*mean ± s.d.*	9.2 ± 1.34	6.8 ± 2.14	8.4 ± 2.00
Writing score—verbal and lexical skills—*mean ± s.d.*	4.6 ± 0.82	3.3 ± 1.31	4.2 ± 1.18
Writing score—spatial orientation—*mean ± s.d.*	4.6 ± 0.71	3.5 ± 1.06	4.3 ± 0.99
Total number of words—*mean ± s.d.*	35.1 ± 12.15	24.4 ± 10.97	31.6 ± 12.78
Percent of words in capital letters—*mean ± s.d.*	8.0 ± 25.90	14.0 ± 32.57	9.9 ± 28.28
Percent of errors—*mean ± s.d.*	1.4 ± 3.31	16.6 ± 25.77	6.4 ± 16.48
Dictated Text			
Total writing score—*mean ± s.d.*	9.4 ± 1.09	7.6 ± 1.87	8.9 ± 1.62
Writing score—verbal and lexical skills—*mean ± s.d.*	4.7 ± 0.75	3.5 ± 1.19	4.3 ± 1.07
Writing score—spatial orientation—*mean ± s.d.*	4.8 ± 0.50	4.2 ± 0.95	4.6 ± 0.73
Total number of errors—*mean ± s.d.*	1.1 ± 2.92	8.7 ± 9.27	3.6 ± 6.75

Results for the evaluation of predictors for cognitive impairment are shown in Table 2. The multivariate analysis confirmed age (OR: 1.07; 95%CI: 1.02–1.13; *p* = 0.008) together with three other (even if not fully significant) characteristics of spontaneous text (writing score - spatial orientation; total number of words; and percent of errors) as independent factors associated with cognitive impairment.

**Table 2:** Logistic regression models evaluating predictors for cognitive impairment (MMSE <24; *N* = 167).

	Univariable analysis		Multivariable analysis		
Variable	OR (95% C.I.)	*p*	β + S.E.	OR (95% C.I.)	*p*
Age (years)	1.11 (1.06–1.15)	<0.001	0.069 ± 0.026	1.07 (1.02–1.13)	0.008
Sex (Male vs. Female)	0.81 (0.40–1.61)	0.54			
Years of education	0.81 (0.74–0.89)	<0.001			
Spontaneous Text					
Writing score—verbal and lexical skills	0.34 (0.24–0.49)	<0.001			
Writing score—spatial orientation	0.26 (0.16–0.40)	<0.001	−0.552 ± 0.293	0.58 (0.32–1.02)	0.059
Total number of words	0.91 (0.88–0.95)	<0.001	−0.044 ± 0.025	0.96 (0.91–1.00)	0.076
Percent of words in capital letters	1.01 (1.00–1.02)	0.21			
Percent of error	1.22 (1.13–1.32)	<0.001	0.133 ± 0.039	1.14 (1.06–1.23)	0.001

A ROC curve was derived, showing high area-under-the-curve (AUC) values: 0.901 (95% CI: 0.853–0.948, *p* < 0.001), indicating good diagnostic performance in predicting the outcome.

The whole sample was then randomly split into a training cohort (*N* = 118) and a validation cohort (*N* = 49) for performing an internal validation of the model (Table 3).

**Table 3:** Internal validation, random selection of cohorts.

	Normal (MMSE ≥ 24)	Cognitive impairment (MMSE < 24)	Total
Training cohort, n (%)	80 (67.8)	38 (32.2)	118 (100.0)
Validation cohort, n (%)	33 (67.3)	16 (32.7)	49 (100.0)

From the training cohort we calculated the coefficients of the multivariable logistic regression model (Table 4).

**Table 4:** Coefficient of multivariable logistic regression model obtained from the training cohort (*N* = 118).

	β
Age (years)	0.085
Spontaneous Text	
Writing score—spatial orientation	−0.654
Total number of words	−0.055
Percent of errors	0.127
Constant	−3.498

Replication of the original regression model on the training cohort confirmed the significance of the same subset of predictors (including those with borderline significance on the whole sample) and the coefficients in Table 4 were used for setting the final equation of the score (**COGITAT**—**COG**nitive **I**mpairment **assessment T**hrough h**A**ndwri**T**ing) and subsequently the probability of having cognitive impairment (P):


$$\begin{array}{l} \mathrm{COGITAT}\;\mathrm{Score}=0.085*\mathrm{AGE}–0.654*\mathrm{WSSO}\\ \;–0.055*\mathrm{WORD}+0.127*\mathrm{PERR} \end{array}$$

$$P=\frac{e^{\left(-3.498+0.085\mathrm{AGE}-0.654\mathrm{WSSO}-0.055\mathrm{WORD}+0.127\mathrm{PERR}\right)}}{1+e^{\left(-3.498+0.085\mathrm{AGE}-0.654\mathrm{WSSO}-0.055\mathrm{WORD}+0.127\mathrm{PERR}\right)}}$$

Where:

P: Probability of having cognitive impairment.

AGE: Age.

WSSO: Writing Score Spatial Orientation.

WORD: Total number of words.

PERR: Percent of errors.

**Figure 8:**
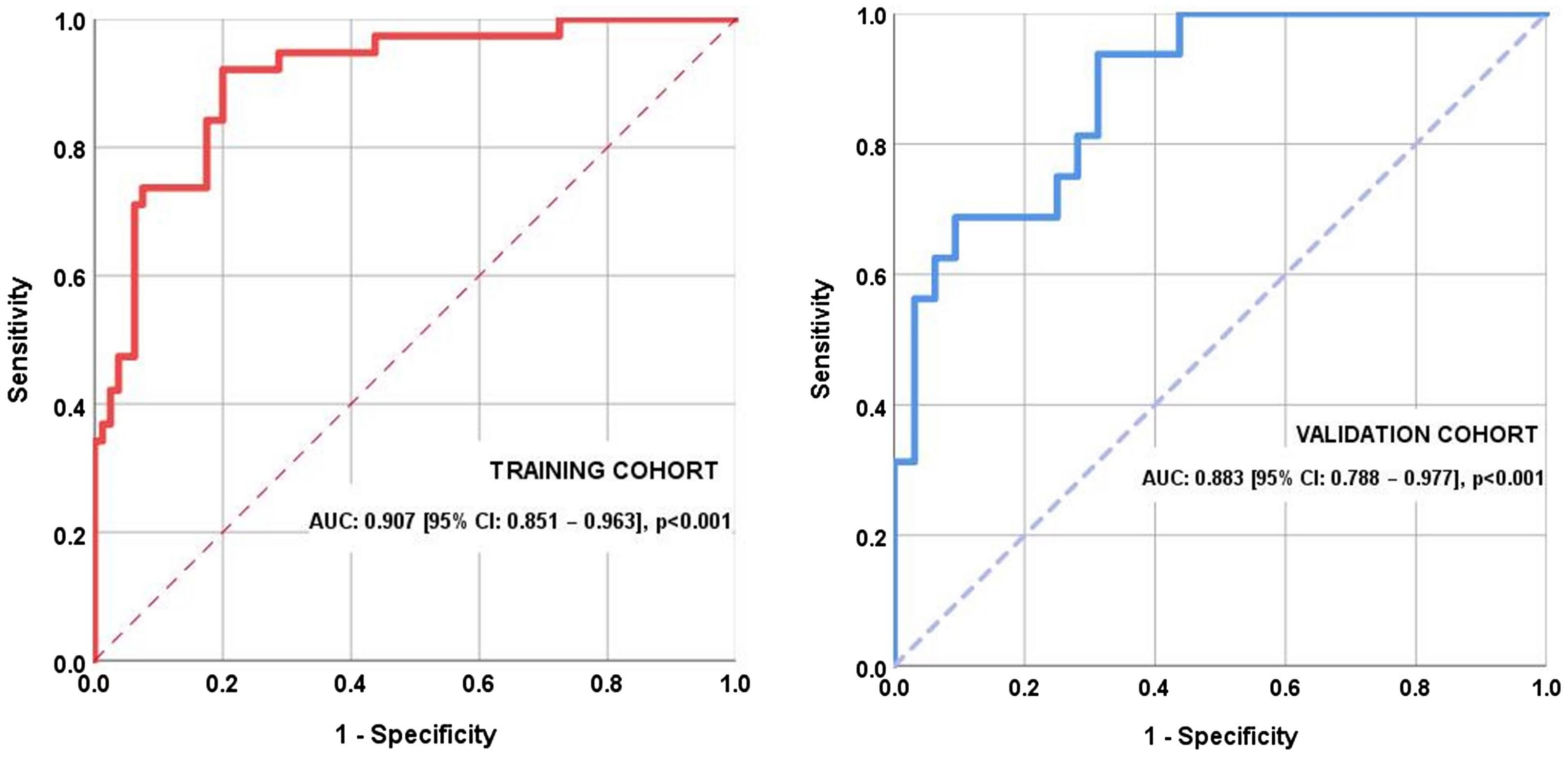
Receiver operating characteristic (ROC) curves for the training and the internal validation cohorts.

The AUC for the training cohort (Figure 8) was 0.907 [95% CI: 0.851–0.963], *p* < 0.001, suggesting a very good predictive performance of the model.

Two optimal cut-off points for the COGITAT score were identified in the training dataset, one that maximizes specificity and the other that favors sensitivity. The first one was found to be 4.258 (specificity 95.0% and sensitivity 47.4%) and patients scoring this high or higher were classified with a 95% probability as being cognitively impaired. The second cut-off score was set to 1.959 (sensitivity 94.7% and specificity 71.2%) and patients scoring this low or lower were classified with a 95% probability as being cognitively normal.

ROC curve graphed in the validation cohort (Figure 8) with the same score showed an AUC of 0.883 [95% CI: 0.788–0.977], *p* < 0.001. The application of the first cut-off produced a sensitivity of 50.0% and a specificity of 96.9%, while the application of the second score produced a sensitivity of 81.2%, a specificity of 28.1%.

To facilitate the clinical application of these findings, an instrument was developed that automatically calculates the score and estimated probability of cognitive impairment after entering the predictor variable data. Higher scores indicate a higher probability of cognitive deterioration. The higher the score compared to the cut-off value, the greater the patient’s risk, and the lower the score compared to the cut-off value, the lower the patient’s risk. The Excel spreadsheet can be downloaded at the following link.^1^

## 4 Discussion

It has been frequently shown that handwriting conveys useful information about the cognitive state of the person who wrote it (Forbes et al., 2004; Croisile, 2005; Shenkin et al., 2008; Renier et al., 2016). However, for clinical or forensic purposes, it is necessary to have an instrument that quantifies the alterations of pathological handwriting, rather than merely describing them. To address this need, some of us earlier created and tested the “writing score” (Fontana et al., 2008; Balestrino et al., 2012), which we briefly summarized above. Unlike other investigations, the writing score provides a numerical value that quantifies the quality of handwriting; thus, it may be used for diagnostic or forensic purposes. In a most fascinating investigation, the writing score has been used to show how King George III of England’s handwriting kept deteriorating during the course of his neuropsychiatric disorder, whose exact nature is still a matter of debate (Peters, 2015).

Our present research is an attempt to further advance the quantitative analysis of handwriting. To do so, we introduced additional parameters and identified two cut-off scores to detect cognitive impairment or normal mental state with high probability.

Thus, we aimed to overcome the problem that previous research on the writing score did not yield a precise cut-off value that could reliably discriminate between normal and cognitively impaired individuals. Preliminary findings suggested that very high or very low scores on the writing score almost certainly indicate that the writer is cognitively normal or cognitively impaired, respectively (Balestrino et al., 2018). Recently, one of us conducted a proof-of-concept investigation in which we suggested that the sensitivity and specificity of the writing score could be improved by including information about how many spelling errors the writer made and how many words he/she wrote (Balestrino, 2022).

In the present investigation, we further advanced our research to identify a novel tool that, starting from the writing score, may be even more useful in identifying cognitively deteriorated people based on their handwriting. To this end, we analyzed parameters that the scientific literature suggests correlate with cognitive deterioration, such as the total number of written words (Henderson et al., 1992), the percent of spelling errors (Silveri et al., 2007), and the percent of words written (totally or partially) in capital letters (Graham, 2000).

We used only MMSE as a gage of cognitive deterioration because it is probably the most widely used test for this purpose, and it has good sensitivity and specificity in identifying cognitive deterioration (Tsoi et al., 2015). Further research is needed to investigate the relationship between the COGITAT score and specific neuropsychological domains, and additional tests investigating specific domains shall be used for this purpose. However, in a forensic validation study akin to ours, the MMSE score was found to correlate in a significant and robust way with the score obtained at a test of financial competency (Giannouli et al., 2018).

In the univariate analysis (Table 2) both age and years of education, but not sex, were different in cognitively impaired people, defined as having MMSE<24 (Measso et al., 1993; Tsoi et al., 2015). Specifically, cognitively impaired subjects were significantly older and had fewer years of education, both findings that were expected based on scientific literature data (LoGiudice and Watson, 2014; Subramaniam et al., 2015). Still in the univariate analysis (Table 2), both subsets of writing score were significantly worse, as expected, in subjects with cognitive deterioration, thus confirming the previous findings by some of us (Fontana et al., 2008; Balestrino et al., 2012). Furthermore, the total number of written words and the percent of spelling errors were significantly different between subjects with cognitive deterioration (MMSE<24) and subjects with normal cognitive status (MMSE≥24), in the sense that cognitively impaired subjects wrote significantly fewer words and made significantly more spelling mistakes, the latter parameters being significantly worse (Table 2). It was expected that those with cognitive impairment would use less words because patients with this condition are known to have lower verbal fluency (Henry et al., 2004). Similarly, the increased percentage of spelling mistakes had been previously reported in cognitively impaired people (Silveri et al., 2007). By contrast, there was no significant difference in the percent of words written in capital letters (Table 2). We offer a possible explanation for this observation by speculating that elderly individuals may employ capital letters to partially mask motor rather than cognitive dysfunction. Further research is needed to possibly confirm this hypothesis.

Then, we carried out a multivariate analysis (Table 2), which confirmed older age and percent of errors as significant predictors of cognitive impairment (*p* < 0.008 and *p* < 0.001, respectively). The “spatial orientation” subset of the writing score had borderline statistical predictive significance (*p* = 0.059), as did the total number of written words (*p* = 0.076). All these parameters were confirmed as significant at validation stage, and therefore, all of them were included for building a single score, which we called the **COG**nitive **I**mpairment assessment **T**hrough h**A**ndwri**T**ing (**COGITAT**) score.

Two different cut-off points for COGITAT score were identified and proved capable of correctly identifying cognitively impaired people and normal people, respectively, with high sensitivity and specificity.

Our study does not allow to assign with the same probability to either normal or altered cognitive status subjects having a COGITAT score between the two above cut-off scores. Further research is needed to possibly overcome this limitation. Nevertheless, we believe that the ability to judge with a statistically acceptable degree of probability a sizable number of subjects may make the COGITAT score a valuable tool in the forensic analysis of disputed wills, a field where judgment is notoriously difficult because at the time of the trial, the testator can no longer be examined, and both health records and witnesses’ reports are often absent or conflicting. Moreover, and perhaps most importantly, a quantitative and statistically sound analysis of handwriting may provide valuable information about the testator’s mental state right at the moment when he/she was writing the will, helping to dispel whatever uncertainty that might arise from the fact that retrospective data such as medical evaluations or testimonies are frequently far away in time from the writing of the will.

In recent years, there has been an increase in scientific interest in using handwriting in the diagnosis of Alzheimer’s Disease (AD), and researchers have investigated this issue even by using machine-based approaches. Among them, Cilia and coworkers found that physical parameters of the movement carried out in either handwriting or drawing may be useful in the early diagnosis of AD (Cilia et al., 2019, 2021a, b, 2022). Those results are of great interest and relevance, however we must not forget that AD is mainly defined by a failure in cognition, while motor, sensory, or coordination deficits are less prominent early in the disease (McKhann et al., 1984, 2011; Albert et al., 2011). Thus, we believe that while machine-analyzed parameters of movement are relevant and interesting, the quantitative analysis of neuropsychological parameters of handwriting is of paramount importance in diagnosing AD based on writing characteristics. From this point of view, the COGITAT score gives utmost importance to spatial orientation of the handwriting, number of written words and percentage of errors in writing, all parameters that are relevant to cognitive deficiency.

Summing up, we suggest that handwriting analysis may be an additional tool for the diagnosis and follow up of dementia in the clinical setting. Although further research will help better defining its strengths and limitations, we believe that the COGITAT score has sufficient statistical soundness to be successfully used to help diagnose cognitive deterioration in both forensic and clinical setting.

## Data availability statement

The original contributions presented in the study are included in the article/Supplementary material, further inquiries can be directed to the corresponding author.

## Ethics statement

Ethical review and approval were not required for the study on human participants in accordance with the local legislation and institutional requirements. The studies were conducted in accordance with the local legislation and institutional requirements. The participants provided their written informed consent to participate in this study.

## Author contributions

MB: Conceptualization, Funding acquisition, Project administration, Supervision, Writing – original draft, Writing – review & editing. AB: Investigation, Writing – review & editing. NG: Investigation, Writing – review & editing. MP: Investigation, Supervision, Validation, Writing – review & editing. CR: Investigation, Writing – review & editing. PA: Writing – review & editing. LC: Investigation, Writing – review & editing. IS: Data curation, Formal analysis, Validation, Writing – original draft.

## Footnotes

^1^https://osf.io/xvt9j/?view_only=68be825d55ae467282d7268f92f06ca4
