# Supplementary material for: Cognitive impairment assessment through handwriting (COGITAT) score: a novel tool that predicts cognitive state from handwriting for forensic and clinical applications
Source: Front Psychol. 2024 Mar 28;15:1275315. doi: 10.3389/fpsyg.2024.1275315 (PMC11007210; doi:10.3389/fpsyg.2024.1275315)
Supplement: Supplementary file 1 [file Table_1.pdf]

Subject number	Group (1=case, 0=caregiver)	Sex (1=male, 0=female)	Age (years)	Education (years)	MMSE	Spontaneous text: Writing score, "verbal and lexical skills" component	Spontaneous text: Writing score, "Spatial orientation" component	Spontaneous text: Writing score, total	Spontaneous text, number of written words	Spontaneous text: number of words written even partially in capital letters	Spontaneous text: percentage of words written even partially in capital letters	Spontaneous text, number of orthographic mistakes	Spontaneous text, percentage of orthographic mistakes/number of words	Dictated text: Writing score, "verbal and lexical skills" component	Dictated text: Writing score, "Spatial orientation" component	Dictated text: Writing score, total	Dictated text, number of orthographic mistakes	Dictated text, percentage of orthographic mistakes/number of words
1	1	1	90	5	8	1	1	2	3	3	100,0	5	167,00	1	1	2	3	5,77
2	1	1	82	5	8	1	1	2	6	0	0,0	2	33,33	1	1	2		0,00
3	1	0	82	5	15	2	2	4	10	9	90,0	4	40,00	2	4	6	18	34,62
4	1	0	83	13	14	2	3	5	9	0	0,0	3	33,33	3	3	6	9	17,31
5	1	1	73	7	6	1	3	4	32	0	0,0	13	40,63	2	4	6	11	21,15
6	1	0	84	2	16	2	3	5	28	0	0,0	7	25,00	2	4	6	17	32,69
7	1	1	78	8	19	2	2	4	20	3	15,0	4	20,00	3	4	7	3	5,77
8	1	0	89	8	16	3	3	6	16	1	6,3	2	12,50	3	4	7	4	7,69
9	1	0	88	5	9	1	2	3	13	0	0,0	0	0,00	2	5	7	12	23,08
10	1	0	65	12	20	4	3	7	29	0	0,0	9	31,03	2	3	5	28	53,85
11	1	0	68	8	19	4	4	8	18	0	0,0	4	22,22	3	4	7	11	21,15
12	1	1	69	8	16	3	4	7	17	17	100,0	3	17,65	2	5	7	29	55,77
13	1	0	76	5	18	3	3	6	20	0	0,0	2	10,00	4	4	8	13	25,00
14	1	0	81	13	21	3	3	6	26	2	7,7	2	7,69	4	4	8	4	7,69
15	0	0	77	8	22	4	5	9	33	0	0,0	7	21,21	4	5	9	3	5,77
16	1	0	80	5	16	3	3	6	29	0	0,0	2	6,90	4	4	8	9	17,31
17	1	0	84	6	16	4	4	8	27	0	0,0	2	7,41	4	4	8	8	15,38
18	1	0	71	8	14	4	2	6	13	0	0,0	0	0,00	4	4	8	3	5,77
19	1	0	82	5	17	4	4	8	28	0	0,0	0	0,00	4	5	9	6	11,54
20	1	1	82	13	22	5	4	9	23	23	100,0	0	0,00	4	3	7	2	3,85
21	1	0	74	15	15	5	4	9	19	0	0,0	0	0,00	5	5	10	0	0,00
22	1	1	85	13	17	5	4	9	37	0	0,0	0	0,00	5	5	10	0	0,00
23	0	0	53	21	22	5	5	10	25	25	100,0	0	0,00	5	5	10	0	0,00
24	1	1	62	8	12	1	2	3	7	2	28,6	5	71,43	1	3	4	46	88,46
25	1	1	86	5	11	1	1	2	14	12	85,7	6	42,86	2	4	6	18	34,62
26	1	0	80	5	11	2	3	5	10	10	100,0	4	40,00	2	5	7	28	53,85
27	1	0	93	1	14	2	3	5	22	0	0,0	7	31,82	2	3	5	16	30,77
28	1	1	81	6	22	3	3	6	25	0	0,0	6	24,00	3	3	6	14	26,92
29	1	0	83	5	12	2	4	6	31	0	0,0	7	22,58	2	5	7	22	42,31
30	1	1	88	11	19	2	3	5	55	0	0,0	15	27,27	3	3	6	9	17,31
31	1	0	92	13	13	2	4	6	19	0	0,0	2	10,53	3	5	8	3	5,77
32	1	1	90	8	22	3	4	7	29	0	0,0	5	17,24	4	5	9	3	5,77
33	1	0	68	5	23	4	3	7	14	0	0,0	3	21,43	4	4	8	8	15,38
34	1	0	87	8	15	4	4	8	24	0	0,0	3	12,50	4	5	9	8	15,38
35	1	0	83	5	20	3	3	6	32	3	9,4	4	12,50	4	5	9	3	5,77
36	1	0	88	7	16	3	3	6	26	0	0,0	0	0,00	4	4	8	4	7,69
37	1	0	62	13	16	3	3	6	17	1	5,9	2	11,76	4	4	8	0	0,00
38	1	0	81	7	23	4	4	8	29	0	0,0	3	10,34	5	5	10	8	15,38
39	1	0	80	5	22	3	4	7	29	0	0,0	2	6,90	2	3	5	19	36,54
40	0	1	85	5	22	4	5	9	26	0	0,0	2	7,69	4	5	9	3	5,77
41	1	0	84	5	10	5	4	9	11	0	0,0	0	0,00	4	4	8	6	11,54
42	1	0	82	17	20	5	3	8	15	0	0,0	0	0,00	5	4	9	0	0,00
43	1	0	83	8	18	5	4	9	23	0	0,0	1	4,35	5	5	10	8	15,38

44	1	0	87	11	22	4	5	9	30	0	0,0	1	3,33	4	5	9	2	3,85
45	1	0	87	8	22	3	5	8	40	0	0,0	1	2,50	4	5	9	4	7,69
46	1	0	75	5	18	4	4	8	27	0	0,0	1	3,70	4	4	8	5	9,62
47	1	0	81	6	17	5	4	9	27	0	0,0	0	0,00	5	4	9	0	0,00
48	1	1	69	8	21	3	5	8	36	2	5,6	3	8,33	3	5	8	11	21,15
49	1	1	75	10	17	4	3	7	36	0	0,0	0	0,00	5	4	9	0	0,00
50	1	1	77	14	14	2	5	7	61	0	0,0	6	9,84	3	5	8	14	26,92
51	1	0	79	8	22	5	5	10	25	0	0,0	0	0,00	5	5	10	0	0,00
52	0	0	76	8	22	5	4	9	34	0	0,0	0	0,00	5	4	9	0	0,00
53	1	0	70	13	19	5	4	9	32	0	0,0	0	0,00	5	5	10	0	0,00
54	0	1	69	8	23	5	5	10	30	0	0,0	0	0,00	4	5	9	5	9,62
55	1	0	89	5	24	2	3	5	20	0	0,0	3	15,00	3	5	8	11	21,15
56	1	1	82	18	24	2	2	4	17	6	35,3	1	5,88	4	5	9	6	11,54
57	0	0	83	4	29	4	4	8	20	0	0,0	2	10,00	4	4	8	2	3,85
58	1	1	79	5	24	4	2	6	24	0	0,0	2	8,33	4	3	7	11	21,15
59	1	1	80	7	27	4	5	9	21	0	0,0	3	14,29	4	5	9	10	19,23
60	1	1	76	10	29	2	3	5	42	3	7,1	4	9,52	2	3	5	15	28,85
61	1	1	79	13	26	3	3	6	26	0	0,0	0	0,00	3	4	7	2	3,85
62	0	0	75	8	29	4	5	9	17	0	0,0	1	5,88	5	5	10	1	1,92
63	1	0	76	10	24	4	4	8	25	0	0,0	1	4,00	4	4	8	2	3,85
64	1	0	86	16	29	5	3	8	33	0	0,0	0	0,00	5	4	9	0	0,00
65	1	0	73	5	29	4	4	8	31	0	0,0	2	6,45	4	4	8	2	3,85
66	1	0	83	9	30	5	5	10	23	0	0,0	0	0,00	5	5	10	0	0,00
67	1	1	66	8	27	4	3	7	20	20	100,0	0	0,00	5	3	8	0	0,00
68	1	1	73	6	26	4	5	9	25	0	0,0	1	4,00	4	5	9	6	11,54
69	0	1	93	13	28	5	4	9	47	47	100,0	0	0,00	5	5	10	1	1,92
70	1	0	82	13	29	5	5	10	27	0	0,0	0	0,00	5	5	10	0	0,00
71	1	1	70	11	26	3	4	7	33	0	0,0	0	0,00	3	4	7	0	0,00
72	1	1	78	13	30	5	4	9	31	0	0,0	0	0,00	5	4	9	0	0,00
73	1	0	78	5	28	4	5	9	37	1	2,7	1	2,70	4	5	9	2	3,85
74	1	0	72	8	26	5	4	9	25	0	0,0	0	0,00	5	5	10	1	1,92
75	1	0	76	8	29	5	5	10	24	24	100,0	0	0,00	5	4	9	0	0,00
76	1	1	71	27	30	5	5	10	19	0	0,0	0	0,00	5	5	10	0	0,00
77	0	0	78	14	30	5	5	10	30	0	0,0	0	0,00	5	5	10	0	0,00
78	1	0	74	13	30	3	5	8	44	0	0,0	1	2,27	3	5	8	0	0,00
79	1	0	85	7	30	5	5	10	43	0	0,0	0	0,00	5	5	10	0	0,00
80	1	0	84	7	26	5	5	10	45	0	0,0	0	0,00	5	5	10	0	0,00
81	0	0	70	10	29	5	5	10	28	0	0,0	0	0,00	5	5	10	0	0,00
82	0	0	69	5	29	5	5	10	28	0	0,0	0	0,00	5	5	10	0	0,00
83	0	0	72	21	30	5	5	10	33	0	0,0	0	0,00	5	5	10	0	0,00
84	0	0	57	13	30	5	5	10	20	0	0,0	0	0,00	5	5	10	0	0,00
85	0	0	59	13	30	5	5	10	23	0	0,0	0	0,00	5	5	10	0	0,00
86	1	0	79	11	28	5	5	10	49	0	0,0	0	0,00	5	5	10	0	0,00
87	0	0	70	17	29	5	5	10	42	0	0,0	0	0,00	5	5	10	0	0,00
88	0	0	66	9	27	5	5	10	37	0	0,0	0	0,00	5	5	10	0	0,00
89	0	0	62	10	30	5	5	10	34	0	0,0	0	0,00	5	5	10	0	0,00
90	0	1	63	6	30	5	5	10	38	0	0,0	0	0,00	5	5	10	0	0,00
91	0	0	53	15	30	5	5	10	29	0	0,0	0	0,00	5	5	10	0	0,00
92	0	0	53	18	28	5	5	10	31	0	0,0	0	0,00	5	5	10	0	0,00
93	0	0	52	17	30	5	5	10	32	1	3,1	0	0,00	5	5	10	0	0,00

94	1	1	64	8	28	5	4	9	54	1	1,9	0	0,00	5	4	9	2	3,85
95	0	0	51	15	30	5	5	10	34	0	0,0	0	0,00	5	5	10	0	0,00
96	0	1	55	8	30	5	5	10	43	0	0,0	0	0,00	5	5	10	1	1,92
97	0	0	50	13	29	5	5	10	37	0	0,0	0	0,00	5	5	10	0	0,00
98	0	1	54	19	30	5	5	10	46	0	0,0	0	0,00	5	5	10	0	0,00
99	0	0	51	20	29	3	5	8	57	0	0,0	0	0,00	4	5	9	1	1,92
100	0	0	67	17	30	5	5	10	78	0	0,0	1	1,28	5	5	10	0	0,00
101	0	0	80	5	28	4	5	9	24	0	0,0	4	16,67	4	5	9	10	19,23
102	1	0	79	5	24	2	5	7	27	0	0,0	2	7,41	2	4	6	15	28,85
103	1	1	81	13	26	3	3	6	34	0	0,0	1	2,94	3	4	7	0	0,00
104	0	1	89	13	29	5	4	9	22	0	0,0	0	0,00	4	4	8	7	13,46
105	0	0	73	8	29	4	4	8	22	0	0,0	1	4,55	5	5	10	0	0,00
106	0	1	86	11	28	5	4	9	26	0	0,0	0	0,00	5	4	9	0	0,00
107	1	1	80	10	29	4	4	8	44	0	0,0	4	9,09	5	5	10	2	3,85
108	0	0	89	5	27	5	5	10	38	0	0,0	2	5,26	5	5	10	3	5,77
109	1	0	84	7	28	5	4	9	28	0	0,0	0	0,00	5	5	10	2	3,85
110	0	0	66	6	27	5	3	8	11	11	100,0	0	0,00	5	4	9	0	0,00
111	1	1	72	13	30	4	4	8	29	1	3,4	0	0,00	5	5	10	0	0,00
112	0	0	79	12	30	5	5	10	37	0	0,0	2	5,41	5	5	10	0	0,00
113	0	0	69	5	30	5	4	9	20	0	0,0	0	0,00	5	4	9	0	0,00
114	0	0	84	14	24	5	5	10	39	0	0,0	1	2,56	5	5	10	2	3,85
115	1	1	81	10	28	5	5	10	31	1	3,2	0	0,00	5	5	10	0	0,00
116	1	0	84	19	30	5	5	10	35	0	0,0	0	0,00	5	5	10	0	0,00
117	0	1	62	9	29	5	3	8	20	0	0,0	0	0,00	5	4	9	0	0,00
118	1	1	85	17	24	3	5	8	59	0	0,0	2	3,39	3	5	8	4	7,69
119	1	1	74	7	30	5	5	10	27	0	0,0	0	0,00	5	5	10	0	0,00
120	1	1	70	13	26	5	5	10	23	23	100,0	0	0,00	5	5	10	0	0,00
121	1	0	80	4	29	5	5	10	36	0	0,0	0	0,00	5	5	10	0	0,00
122	0	1	77	17	30	5	5	10	34	0	0,0	0	0,00	5	5	10	0	0,00
123	1	0	77	10	24	5	5	10	34	3	8,8	0	0,00	5	4	9	0	0,00
124	0	0	50	13	30	4	5	9	26	0	0,0	2	7,69	5	5	10	0	0,00
125	0	1	67	18	26	5	5	10	28	0	0,0	0	0,00	5	5	10	0	0,00
126	0	1	71	13	28	5	5	10	34	34	100,0	0	0,00	5	5	10	0	0,00
127	1	0	80	8	30	5	4	9	52	0	0,0	0	0,00	5	4	9	0	0,00
128	1	0	77	13	30	5	5	10	44	0	0,0	0	0,00	5	5	10	0	0,00
129	0	0	69	8	25	5	5	10	34	0	0,0	0	0,00	3	5	8	2	3,85
130	0	0	77	13	26	5	5	10	45	0	0,0	0	0,00	5	5	10	0	0,00
131	0	0	54	11	30	5	5	10	24	24	100,0	1	4,17	5	5	10	0	0,00
132	0	0	68	19	30	5	5	10	34	0	0,0	0	0,00	5	5	10	0	0,00
133	0	0	63	8	28	5	5	10	28	0	0,0	0	0,00	5	5	10	0	0,00
134	0	0	59	18	26	5	5	10	24	3	12,5	0	0,00	5	5	10	0	0,00
135	1	0	71	10	29	5	5	10	41	0	0,0	0	0,00	5	5	10	0	0,00
136	1	0	71	19	30	5	5	10	42	0	0,0	0	0,00	5	5	10	0	0,00
137	0	1	57	13	27	5	5	10	24	0	0,0	0	0,00	5	5	10	0	0,00
138	1	0	68	10	28	4	4	8	56	0	0,0	1	1,79	5	5	10	0	0,00
139	0	0	50	8	30	5	5	10	19	0	0,0	0	0,00	5	5	10	0	0,00
140	1	0	60	19	30	5	5	10	32	1	3,1	0	0,00	5	5	10	0	0,00
141	0	1	75	13	25	5	5	10	52	0	0,0	0	0,00	5	5	10	0	0,00
142	0	0	57	13	30	5	5	10	29	0	0,0	0	0,00	5	5	10	0	0,00
143	0	0	56	11	30	5	5	10	30	0	0,0	0	0,00	5	5	10	0	0,00

144	0	1	55	18	30	5	5	10	30	1	3,3	0	0,00	5	5	10	0	0,00
145	0	0	52	13	30	5	5	10	27	0	0,0	0	0,00	5	5	10	0	0,00
146	0	1	57	18	28	5	4	9	40	0	0,0	0	0,00	5	5	10	0	0,00
147	0	0	61	12	30	5	5	10	39	1	2,6	0	0,00	5	5	10	0	0,00
148	0	0	56	13	30	5	5	10	33	0	0,0	0	0,00	5	5	10	0	0,00
149	0	1	63	21	30	5	5	10	44	0	0,0	0	0,00	5	5	10	0	0,00
150	0	0	56	18	30	5	5	10	35	0	0,0	0	0,00	5	5	10	0	0,00
151	0	0	70	13	30	4	5	9	60	0	0,0	0	0,00	4	5	9	0	0,00
152	0	1	53	8	30	5	5	10	33	0	0,0	0	0,00	5	5	10	0	0,00
153	0	1	57	18	30	5	5	10	39	0	0,0	0	0,00	5	5	10	0	0,00
154	0	0	54	10	30	5	5	10	36	36	100,0	0	0,00	5	5	10	0	0,00
155	0	0	57	13	26	5	5	10	40	2	5,0	0	0,00	4	4	8	3	5,77
156	0	0	60	23	30	5	5	10	46	0	0,0	0	0,00	5	5	10	0	0,00
157	0	1	59	14	30	5	5	10	45	0	0,0	0	0,00	5	5	10	0	0,00
158	1	0	79	9	29	5	5	10	71	0	0,0	0	0,00	5	5	10	0	0,00
159	0	1	54	18	30	5	5	10	40	0	0,0	0	0,00	5	5	10	0	0,00
160	0	0	52	13	30	5	5	10	40	1	2,5	0	0,00	5	5	10	0	0,00
161	0	0	50	22	30	5	5	10	40	0	0,0	0	0,00	5	5	10	0	0,00
162	0	1	53	18	29	5	5	10	46	0	0,0	0	0,00	5	5	10	0	0,00
163	0	1	54	15	28	5	5	10	49	1	2,0	0	0,00	5	5	10	1	1,92
164	0	0	58	12	26	5	5	10	56	0	0,0	0	0,00	5	5	10	0	0,00
165	1	1	58	10	27	4	5	9	68	0	0,0	0	0,00	5	5	10	0	0,00
166	0	0	50	10	26	5	5	10	57	0	0,0	1	1,75	5	5	10	0	0,00
167	1	1	64	7	29	1	3	4						1	3	4		0,00
