# Supplementary material for: Cognitive impairment assessment through handwriting (COGITAT) score: a novel tool that predicts cognitive state from handwriting for forensic and clinical applications
Source: Front Psychol. 2024 Mar 28;15:1275315. doi: 10.3389/fpsyg.2024.1275315 (PMC11007210; doi:10.3389/fpsyg.2024.1275315)
Supplement: Supplementary file 2 [file Table_2.pdf]

Supplementary table II - The writing score[1,2]

VERBAL AND LEXICAL SKILLS

**EXAMINER'S EVALUATION**	**SCORE**
No mistakes	**5**
Some mistakes (for example, missing or wrong letters, words written in a wrong way), however the text can be easily understood.	**4**
Some mistakes (as above), however the text can be understood with some effort.	**3**
Some mistakes (as above), the text can be understood only with considerable effort.	**2**
Text not understandable.	**1**

SPATIAL ORIENTATION

**EXAMINER'S EVALUATION**	**SCORE**
Normally oriented rows. In each row, beginning and end correspond to the page margins.	**5**
Rows slightly distorted or with beginning and end bearing little correspondence to the page margins.	**4**
Rows clearly distorted or with beginning and end not corresponding to the page margins.	**3**
Words or letters inserted where they do not belong in the text.	**2**
Chaotic orientation of the rows.	**1**

1. Fontana, P.; Dagnino, F.; Cocito, L.; Balestrino, M. Handwriting as a Gauge of Cognitive Status: A Novel Forensic Tool for Posthumous Evaluation of Testamentary Capacity. *Neurol Sci* **2008**, *29*, 257–261, doi:10.1007/s10072-008-0977-3.
2. Balestrino, M.; Fontana, P.; Terzuoli, S.; Volpe, S.; Inglese, M.L.; Cocito, L. Altered Handwriting Suggests Cognitive Impairment and May Be Relevant to Posthumous Evaluation. *J Forensic Sci* **2012**, *57*, 1252–1258, doi:10.1111/j.1556-4029.2012.02131.x.
